# Three-dimensional evaluation of pharyngeal airway and maxillary arch in mouth and nasal breathing children with skeletal Class I and II

**DOI:** 10.1186/s12903-022-02355-3

**Published:** 2022-08-01

**Authors:** Janvier Habumugisha, Shu-Yu Ma, Amin S. Mohamed, Bo Cheng, Min-Yue Zhao, Wen-Qing Bu, Yu-Cheng Guo, Rui Zou, Fei Wang

**Affiliations:** 1 Key Laboratory of Shaanxi Province for Craniofacial Precision Medicine Research, Clinical Research Center of Shaanxi Province for Dental and Maxillofacial Diseases, College of Stomatology, Xi’an Jiaotong University, Xi’an, 710004 Shaanxi People’s Republic of China; 2 Department of Orthodontics, Xi’an Jiaotong University, Xi’an, People’s Republic of China

**Keywords:** Mouth breathing, Cone-beam computed tomography, Pharynx, Maxilla

## Abstract

**Objective:**

This study aimed to investigate whether the subjects with mouth breathing (MB) or nasal breathing (NB) with different sagittal skeletal patterns showed different maxillary arch and pharyngeal airway characteristics.

**Methods:**

Cone-beam computed tomography scans from 70 children aged 10 to 12 years with sagittal skeletal Classes I and II were used to measure the pharyngeal airway, maxillary width, palatal area, and height. The independent t-test and the Mann–Whitney U test were used for the intragroup analysis of pharyngeal airway and maxillary arch parameters.

**Results:**

In the Skeletal Class I group, nasopharyngeal airway volume (*P* < 0.01), oropharyngeal airway volume (OPV), and total pharyngeal airway volume (TPV) (all *P* < 0.001) were significantly greater in subjects with NB than in those with MB. Furthermore, intermolar width, maxillary width at the molars, intercanine width, maxillary width at the canines, and palatal area were significantly larger in subjects with NB than in those with MB (all *P* < 0.001). In the Skeletal Class II group, OPV, TPV (both *P* < 0.05) were significantly greater in subjects with NB than in those with MB. No significant differences in pharyngeal airway parameters in the MB group between subjects with Skeletal Class I and those with Skeletal Class II.

**Conclusion:**

Regardless of sagittal Skeletal Class I or II, the pharyngeal airway and maxillary arch in children with MB differ from those with NB. However, the pharyngeal airway was not significantly different between Skeletal Class I and II in children with MB.

## Introduction

Adenotonsillar hypertrophy, allergic and chronic rhinitis, infections, congenital nasal anomalies, polyps, and tumors are risk factors for upper airway obstruction [1, 2]. Consequently, a functional imbalance causes an oral breathing pattern that may affect the facial and dental morphology, leading to malocclusion [2, 3]. According to Melvin Moss' functional matrix theory, soft tissues dictate the growth of the craniofacial complex. Moreover, nasal breathing could aid proper craniofacial growth in conjunction with other functions such as chewing and swallowing [4]. In contrast, mouth breathing affects the proper growth of the craniofacial region [2]. Bresolin et al. [5] evaluated participants with mouth breathing and compared them to controls. They discovered that subjects with mouth breathing had a narrower face, jaw retroposition, protrusion of upper incisors, and a stepper mandibular plane. They concluded that mouth breathers could have a distinct face development pattern than nasal breathers. In some reports, the impact of mouth breathing on craniofacial growth has been a controversial topic. However, a classic primate experiment by Harvold et al. demonstrated that switching from nasal to oral breathing can affect dentofacial growth. In their study, they blocked monkeys’ nostrils with silicon nose plugs. The animals adapted to the nasal obstruction, resulting in narrowing dental arches, open bite tendency, and increases in lower facial height associated with mandibular downward rotation [6].

Understanding the interaction between the pharyngeal airway and skeletal malocclusion has recently received much attention from researchers. They looked at the pharyngeal airway spaces in people with different and vertical skeletal configurations of nasal breathing individuals [3, 7–12]. These studies discovered inconsistent findings on how malocclusion affects the diameters of the upper airway. Claudino et al. [3] reported that the velopharynx and oropharynx airway regions were significantly smaller in Sagittal Skeletal Class II individuals than in Class I and III participants. Oropharyngeal space was less in patients with skeletal Class II than in those with skeletal Class I, according to recent cephalometric research by Gholinia et al. [12]. However, some researchers asserted that the sagittal relationship of the upper and lower jaws does not affect the airway size [11, 13]. Ceylan and Oktay stated that pharyngeal structures underwent postural modifications as the skeletal anteroposterior relationship changed, and therefore, the airway diameter remained unchanged [10]. However, it is crucial to highlight that using lateral cephalometric X-rays to examine the pharyngeal airway might be a limitation of some of these studies. Cephalometric X-rays give two-dimensional linear measurements that are inadequate for precisely estimating the airway volume [14].

Some authors hypothesized that changes in craniofacial development caused by mouth breathing and other oral habits lead to changes in the maxilla and mandible morphology [15, 16]. The study of the morphologic differences of these structures in people with various skeletal malocclusions and different breathing modes might lead to a better understanding of their anatomical variation in these people. Clinically, It might provide additional information for orthodontic diagnosis and treatment planning.

The study casts were the main assessment method in most investigations that evaluated maxillary transverse width and palatal morphology changes in mouth-breathing subjects [17, 18]. However, transverse discrepancies, such as skeletal crossbite and skeletal maxillary width, are more easily identified with cone-beam computed tomography (CBCT) than with study casts [19]. CBCT has a lower radiation dosage than multislice computed tomography in identifying the pharyngeal airway and craniofacial structures. Therefore, CBCT has been suggested to be the best method for recognizing the pharyngeal airway and craniofacial structures [3, 20]. Additionally, CBCT displays the airway better in 3D than 2D cephalometrics [20].

Although some research attempted to analyze airways in people with different sagittal relationships using three-dimensional (3D) evaluation, these studies primarily focused on individuals with nasal breathing (NB) pattern [7, 9, 19, 21]. The relationship between pharyngeal airway and skeletal malocclusion in mouth breathing subjects was not extensively studied in previous literature. Therefore, this study investigated whether subjects with MB and those with NB with different sagittal facial patterns show different maxillary arch and pharyngeal airway characteristics.

## Material and method

### Study design

This retrospective, cross-sectional and observational study was approved by the ethics board committee of Xi’an Jiaotong University's stomatological hospital, ethical approval number:Xjkqll [2018] No.17.

### Sample size calculation

Pandis et al. power calculations formula was used to determine the sample size [22] to detect a difference of at least 2576.61 mm^3^ of the oropharyngeal airway between the two groups (MB and NB) using the standard deviation from a previous study by Alves et al. [23]. At least eight participants in each subgroup were required to achieve this at (α = 0.05, power of 80%).

All patient records from the orthodontic department of Xi’an Jiaotong University's stomatological hospital between January 2018 and November 2021 were screened for inclusion in this cross-sectional retrospective study. For our study, we utilized a sample of 70 CBCT scans of patients (35 males and 35 females) that matched the inclusion criteria.

### Inclusion and exclusion criteria

The inclusion criteria were as follows: (1) age ranging between 10 and 12 years; (2) no previous orthodontic therapy or orthognathic surgery; (3) Skeletal Class I or Skeletal Class II malocclusions (SNA [sella, nasion, A point] angle > 78.8°and < 85.8°, Skeletal Class I with an ANB angle ≥ 1° and ≤ 5°, and Skeletal Class II with an ANB angle > 5°); (4) normal divergence (GoGn-SN angle > 27.3° and < 35.5°, Frankfort mandibular plane angle(FMA°) > 25.32° and < 33.16°) [24], and (5) a normal body mass index (18.5–24 kg/m^2^) [25]. The exclusion criteria were as follows: (1) the presence of syndromes; (2) craniofacial anomalies (cleft lip and palate) or other growth disturbances; (3) subjects with clinically diagnosed posterior crossbites; (4) subjects with an enlarged tongue or ankyloglossia; (5) unclear or absent landmarks on CBCT scans; and (4) the presence of artifacts or distortion on the CBCT scan.

### Selection of mouth and nasal breathing subjects

As per our previous study [25], breathing mode was evaluated by an Orthodontist and an Otolaryngologist. History taking by the Orthodontist was performed. The parents of the children were asked about their children’s sleeping habits, such as if they sleep with their mouths open, the children’s habitual lip position was examined, and the Glatzer mirror test was conducted to detect mouth breathers. A thorough physical examination by an experienced otorhinolaryngologist included rhinomanometry, anterior rhinoscopy, flexible nasopharyngoscopy, or nasopharyngeal x-ray to evaluate nasal airflow and pressure while breathing, as well as pharyngeal airway obstruction. The otorhinolaryngologist and orthodontists categorized the participants as nasal or mouth breathers only after completing these examinations [15, 18].

### CBCT scanning

All CBCT scanning protocols were done using a standard procedure on a cone beam machine (i-CAT; Imaging Sciences International, Hatfield, PA, USA) (120 kV, 5 mA, 1417-cm field of view, 0.4-mm voxels, and scan duration of 8.9 s). Each patient sat with the Frankfort horizontal plane parallel to the floor. During the acquisition of CBCT images, all patients were instructed to hold their breath at the end of expiration and not to swallow with their jaw in maximum intercuspation [25].

**Fig. 1:**
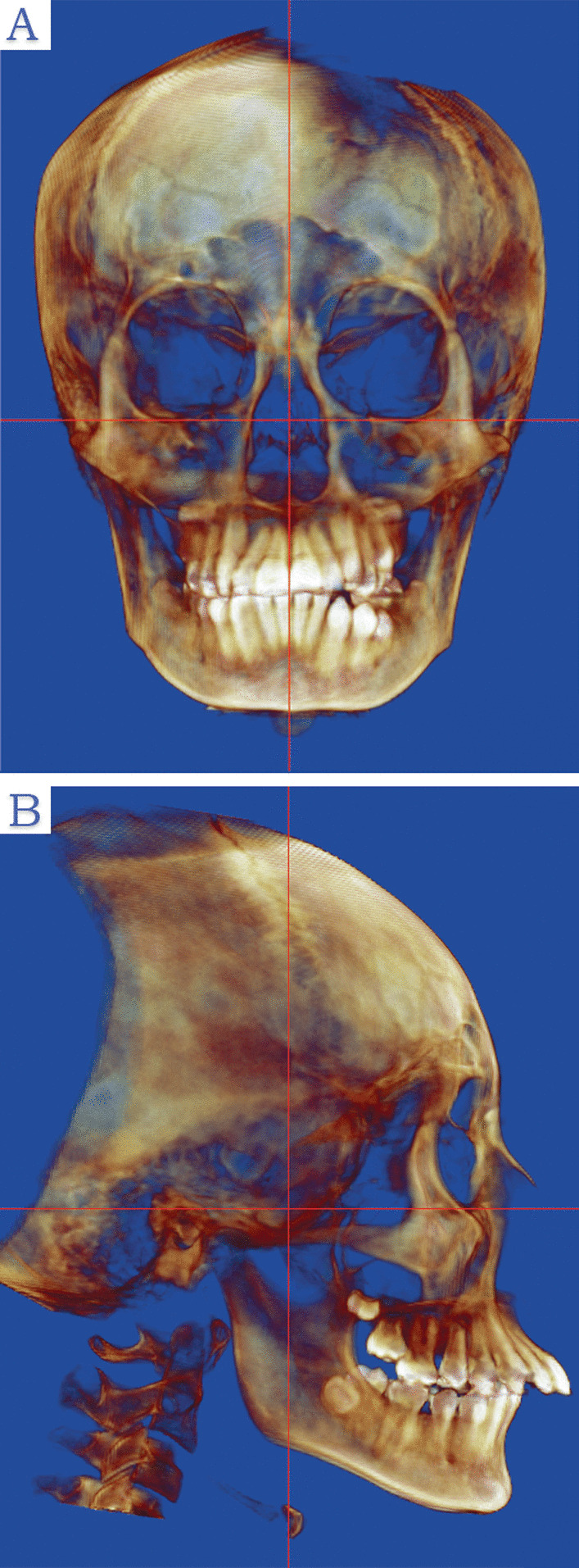
Orientation of an i-CAT image for airway measurement. **A** Frontal view; **B** right sagittal view

The CBCT data were saved in digital imaging and communications in medicine (DICOM)format. All measurements were taken by experienced and trained orthodontists using Dolphin Imaging software (Version 11.7; Dolphin Imaging & Management Solutions, Chatsworth, CA, USA). To avoid the risk of bias, the investigators were blinded to the subject's demographic features. Each digital DICOM formatted image was orientated parallel to the Frankfurt horizontal plane to assess the airway (Fig. 1).

### CBCT Scan orientation and measurements

The axial plane was defined by three points: the right porion, the right orbitale, and the left orbitale. Two reference images were generated to verify that the skull was oriented to the Frankfort plane. The horizontal reference line was established using the porion and the right orbitale in the right sagittal view. The frontal view drew a horizontal reference line between the right and left orbitales. The vertical reference line was formed using the anterior nasal spine and the nasion [9] (Fig. 1). The airway sensitivity level governs the program's capacity to identify differences in grayscale resolution; it was set at 73 for optimal recognition of the airway; Alves et al. assert that a sensitivity threshold of 73 provides the best accurate way to measure airway volume [26]

The pharyngeal airway was divided into two sections (oropharyngeal and nasopharyngeal regions). The volume and area of each were calculated. The total airway volume was considered as the sum of the oropharyngeal and nasopharyngeal regions [9].

**Fig. 2:**
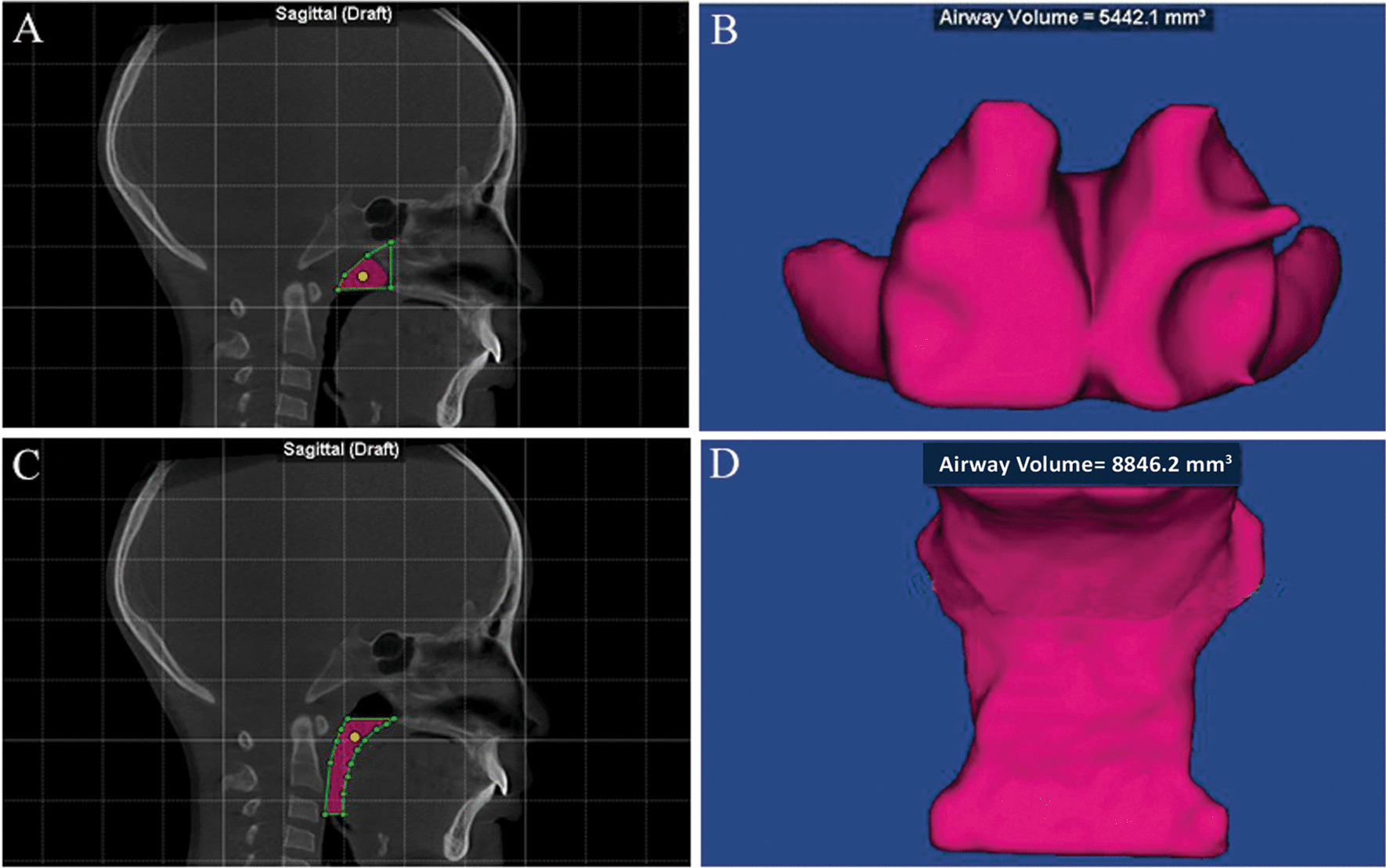
Landmarks for the pharyngeal airway. **A** boundary of the OPV; **B** oropharyngeal airway volume; **C** boundary of the NPV; **D** nasopharyngeal airway volume

**Fig. 3:**
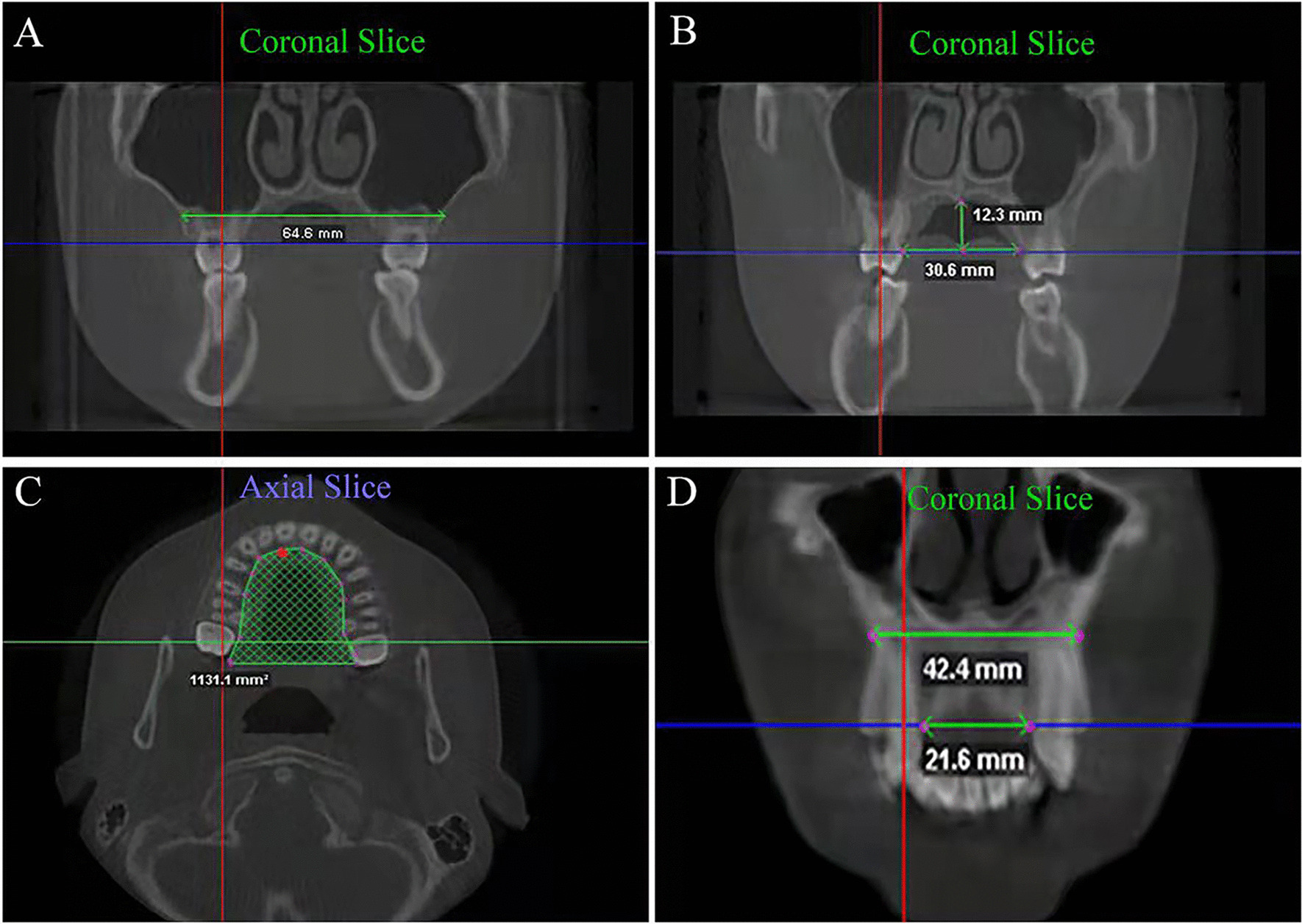
Schematic diagrams of maxillary arch measurements. **A** MWM; **B** IMW and PH; **C** PA; **D** MWC and ICW

The airway volumes were also determined using the seed points, and airway extremities were determined using the landmarks (Fig. 2) and Table 1. The schematic diagrams of maxillary arch measurements are shown in Table 1 and Figs. 3 [27]. The cephalometric scans were formed from CBCT.

**Table 1:** Definitions of pharyngeal airway and maxillary arch parameters

Abbreviations	Definition and illustration
NPV	Nasopharyngeal airway volume: the volume between the superior border of the OPV, which was defined as the line from the posterior nasal spine perpendicular to the top limit of the OPV continuing superiorly to meet the posterior wall, and the outline of the pharynx (Fig. 2A, B)
NPA	Nasopharyngeal airway area: the area between the upper and lower limits of nasopharynx.Once the NPV border was established and the NPV computed, the nasopharyngeal airway area was calculated and shown automatically
OPV	The oropharyngeal airway volume (OPV) was identified as the volume of the pharynx between a line parallel to Frankfurt horizontal plane (FHP) crossing through the posterior nasal spine and another line parallel to FHP at the level of the tip of the epiglottis (Fig. 2C, D)
OPA	Oropharyngeal airway area: the area between the upper and lower limits of oropharynx,Once the OPV border was established and the OPV computed, the oropharyngeal airway area was calculated and shown automatically
TPV	Total volume of the pharyngeal airway: the nasopharyngeal volume plus the oropharyngeal airway volume
MWM	Maxillary width at the molars: a dimension between the deepest points of the bilateral posterior concavities in the maxilla (Fig. 3A)
IMW	Intermolar width: the dimension between the lingual alveolar bone crests of the maxillary bilateral first molars (Fig. 3B)
PH	Palatal height: the vertical dimension from the center of the IMW to the palate’s uppermost point (Fig. 3B)
PA	Palatal area: the area of the palate before the alveolar ridge boundaries from the bilateral maxillary first molars (Fig. 3C)
MWC	Maxillary width at the canines: the dimension between the deepest points of the bilateral maxillary middle concavities at the canines (Fig. 3D)
ICW	Intercanine width: the dimension between the lingual alveolar bone crests of the bilateral canines. (Fig. 3D)

### Statistical analysis

To examine intra and inter-investigator errors, we randomly chose 15 CBCT scans that were measured and compared at baseline and then re-measured 2 weeks later. For intra- and inter-reliability testing, Pearson correlation (r) was utilized. Dahlberg's formula [28] was used to determine the method error $SE = \sqrt {\sum \frac{{D^{2} }}{2N}}$,Where n is the number of subjects and d is the difference between the first and second measurements. All measurements were assessed for normal distribution by the Shapiro–Wilks test. The data were analyzed by IBM SPSS 23 (IBM Corp., Armonk, NY, USA). The chi-square test was used to analyze the distribution of sex between children with MB and those with NB in classes I and II. The independent t-test was used to test differences by age and craniofacial parameters between NB and MB. Moreover, the independent t-test was used for intragroup comparison of pharyngeal airway and maxillary arch parameters, except for parameters of nasopharyngeal airway volume (NPV), the total volume of the pharyngeal airway (TPV), maxillary width at the canines (MWC), and intercanine width (ICW), which were not normally distributed. These non-normally distributed parameters were analyzed by the non-parametric Mann–Whitney U test.

## Results

Intrarater reliability for the first examiner (orthodontist 1, J.H) ranged from 0.92 to 0.99, and the second examiner (orthodontist 2, A.M) ranged from 0.91 to 0.99. Interrater reliability for all measurements ranged from 0.90 to 0.99. The highest volumetric error was 51.65 mm^3^, and the highest error for area measurement was 6.9 mm^2^. The highest linear measurement error was 0.37 mm, while the highest angular measurement error was 0.12°. These findings validated the measurement method’s reproducibility and reliability.

The baseline demographics of sex and age and the various angles in children with MB and those with NB with Skeletal Class I or Skeletal Class II are shown in (Table 2).

**Table 2:** Baseline demographics and various angles in the subjects

	70 samples from 1350							
Descriptives	Class I (n = 34)				Class II (n = 36)			
	MB (n = 17)	NB (n = 17)	TOTAL (n = 34)	*P*	MB (n = 18)	NB (n = 18)	TOTAL (n = 36)	*P*
Males (n)	9 (26.5%)	9 (26.5%)	18 (53%)	0.634^a^	8 (22%)	9 (25%)	17 (47%)	0.738^a^
Females (n)	8 (23.5%)	8 (23.5%)	16 (47%)		10 (28%)	9 (25%)	19 (53%)	
Age (years)	11.12 ± 0.69	11.4 ± 0.71	11.26 ± 0.7	0.232^b^	10.94 ± 0.80	10.56 ± 1.04	10.75 ± 0.92	0.218^b^
SN-GOGN (°)	32.41 ± 2.47	31.82 ± 3.8	32.11 ± 3.13	0.59^b^	33.52 ± 2.47	32.62 ± 2.99	33.07 ± 2.73	0.332^b^
ANB (°)	2.93 ± 1.23	2.47 ± 1.06	2.7 ± 1.14	0.261^b^	7.20 ± 1.52	6.48 ± 0.78	6.84 ± 1.11	0.082^b^
SNA (°)	81.2 ± 4.5	80.43 ± 4.3	80.81 ± 4.4	0.603^b^	82.97 ± 3.32	82.03 ± 3.02	82.5 ± 3.17	0.379^b^
FMA (°)	28.54 ± 3.03	29.30 ± 3.94	28.92 ± 3.48	0.531^b^	29.95 ± 3.66	29.58 ± 3.10	29.76 ± 6.76	0.748^b^

^a^Chi-square test

^b^Independent t-test

**Fig. 4:**
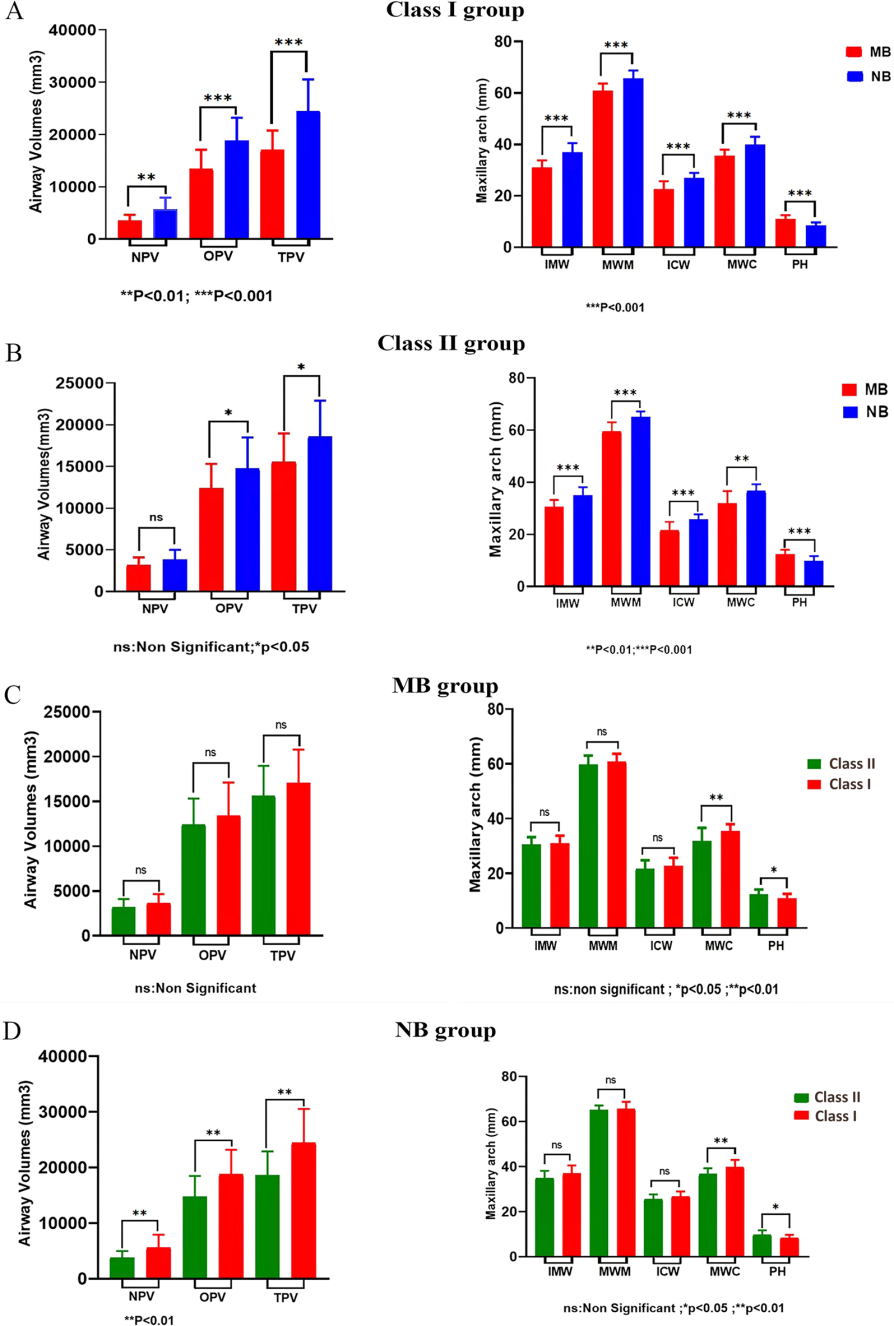
Differences in the pharyngeal airway and maxillary arch by groups. **A** Comparison of MB and NB in the skeletal Class I group; **B** comparison of MB and NB in the skeletal Class II group; **C** comparison of subjects with skeletal Classes I and II in the MB group; **D** comparison of skeletal Classes I and II in the NB group

There were significant differences in the pharyngeal airway and maxillary arch measurements between subjects with MB and those with NB (Table3 and Fig. 4A, B). In the Skeletal Class I group, NPV, OPA (both *P* < 0.01), nasopharyngeal airway area (NPA), OPV, and TPV (all *P* < 0.001) were significantly greater in subjects with NB than in those with MB. Furthermore, IMW, maxillary width at the molars (MWM), ICW, MWC, and PA were significantly larger in subjects with NB than in those with MB (all *P* < 0.001). However, subjects with MB had a significantly greater palatal height than those with NB (*P* < 0.001).

**Table 3:** Analyses between subjects with MB and NB grouped by skeletal classes I and II, and between subjects with skeletal classes I and II grouped by MB and NB

VARIABLES	Skeletal Class I			Skeletal Class II			MB			NB		
	MB (n = 17)	NB (n = 17)	*P*	MB (n = 18)	NB (n = 18)	P	Class I (n = 17)	Class II (n = 18)	*P*	Class I (n = 17)	Class II (n = 18)	*P*
	M ± SD	M ± SD		M ± SD	M ± SD		M ± SD	M ± SD		M ± SD	M ± SD	
NPV(mm^3^)	3626.63 ± 1028.87	5661.98 ± 2269.91	0.002**^b^	3194.71 ± 903.02	3843.31 ± 1158.02	0.069^b^	3626.63 ± 1028.87	3194.71 ± 903.02	0.195^b^	5661.98 ± 2269.91	3843.31 ± 1158.02	0.005**^b^
NPA(mm^2^)	115.71 ± 40.73	199.92 ± 44.93	0.000***^a^	105.61 ± 29.01	149.36 ± 44.22	0.001**^a^	115.71 ± 40.73	105.61 ± 29.01	0.402^a^	199.92 ± 44.93	149.36 ± 44.22	0.002**^a^
OPV(mm^3^)	13,414.95 ± 3689.61	18,838.41 ± 4367.04	0.000***^a^	12,394.61 ± 2919.81	14,750.96 ± 3741.34	0.043*^a^	13,414.95 ± 3689.61	12,394.61 ± 2919.81	0.369^a^	18,838.41 ± 4367.04	14,750.96 ± 3741.34	0.005**^a^
OPA(mm^2^)	480.74 ± 103.21	603.48 ± 84.94	0.001**^a^	423.66 ± 135.85	517.41 ± 164.03	0.071^a^	480.74 ± 103.21	423.66 ± 135.85	0.173^a^	603.48 ± 84.94	517.41 ± 164.03	0.062^a^
TPV(mm^3^)	17,041.58 ± 3741.72	24,500.40 ± 6035.87	0.000***^b^	15,589.32 ± 3368.72	18,594.27 ± 4303.40	0.026*^b^	17,041.58 ± 3741.72	15,589.32 ± 368.72	0.236^b^	24,500.4 ± 6035.87	18,594.2 ± 4303.40	0.002**
MWM(mm)	60.84 ± 2.79	65.67 ± 3.09	0.000***^a^	59.69 ± 3.31	65.07 ± 2.04	0.000***^a^	60.84 ± 2.79	59.69 ± 3.31	0.276^a^	65.67 ± 3.09	65.07 ± 2.04	0.498^a^
IMW(mm)	31.07 ± 2.69	36.90 ± 3.59	0.000***^a^	30.53 ± 2.68	34.99 ± 3.12	0.000***^a^	31.07 ± 2.69	30.53 ± 2.68	0.555^a^	36.90 ± 3.59	34.99 ± 3.12	0.102^a^
PH(mm)	10.98 ± 1.51	8.43 ± 1.26	0.000***^a^	12.45 ± 1.64	9.80 ± 1.88	0.000***^a^	10.98 ± 1.51	12.45 ± 1.64	0.010*^a^	8.43 ± 1.26	9.80 ± 1.88	0.017*^a^
MWC(mm)	35.53 ± 2.44	39.80 ± 3.17	0.000***^b^	31.92 ± 4.68	36.72 ± 2.53	0.001**^b^	35.53 ± 2.44	31.92 ± 4.68	0.008**^b^	39.80 ± 3.17	36.72 ± 2.53	0.03*^b^
ICW(mm)	22.67 ± 3.01	26.80 ± 2.17	0.000***^b^	21.62 ± 3.19	25.68 ± 1.98	0.000***^b^	22.67 ± 3.01	21.62 ± 3.19	0.326^b^	26.80 ± 2.17	25.68 ± 1.98	0.121^b^
PA(mm^2^)	727.79 ± 82.95	987.22 ± 126.52	0.000***^a^	661.77 ± 105.63	919.95 ± 114.67	0.000***^a^	727.79 ± 82.95	661.77 ± 105.63	0.049*^a^	987.22 ± 126.52	919.95 ± 114.67	0.108^a^

*M ± SD* mean ± standard deviation, *MB* mouth breathing, *NB* nasal breathing

^*^P < 0.05; **P < 0.01; ***P < 0.001

^a^Independent T test

^b^Mann Whitney U test

In the Skeletal Class II group, OPV, TPV (both *P* < 0.05), and NPA (*P* < 0.01) were significantly greater in subjects with NB than in those with MB. MWM, IMW, ICW, PA (all *P* < 0.001), and MWC (*P* < 0.01) were significantly greater in subjects with NB than in those with MB. However, subjects with MB had a significantly higher palatal height than those with NB (*P* < 0.001).

Table 3 and Fig. 4C, D compare pharyngeal airway and maxillary arch parameters between Skeletal Class I and II according to the breathing mode. There were no significant differences in pharyngeal airway parameters in the MB group between subjects with Skeletal Class I and those with Skeletal Class II. However, NPV, NPA, OPV, and TPV were significantly greater in subjects with Skeletal Class I than in those with Skeletal Class II in the NB group (all *P* < 0.01).

With regard to maxillary arch measurements, MWC (*P* = 0.01) and PA (*P* < 0.05) were larger in subjects with Skeletal Class I than in those with Skeletal Class II in the MB group. The palatal height was greater in subjects with Skeletal Class II than in those with Skeletal Class I in the MB group.

MWC (*P* < 0.05) was larger in subjects with Skeletal Class I than in those with Skeletal Class II in the MB group. However, subjects with Skeletal Class II had a greater palatal height than those in Skeletal Class I in the NB group (*P* < 0.05).

## Discussion

Previous 3D studies of the pharyngeal airway or maxillary width were only on individuals with a normal breathing mode according to different facial patterns [3, 9, 19]. The difference in pharyngeal airway parameters between individuals with mouth breathing (MB) and those with nasal breathing (NB) with only Skeletal Class I was also assessed [23]. In Chung et al. study, the skeletal facial patterns were not examined in their study subjects [29]. There are still gaps in knowledge in investigations of the pharyngeal airway or maxillary dimension comparing oral and nasal breathing patients with different sagittal Skeletal patterns.

In the current study, no significant differences were found in pharyngeal airway measurements between subjects with Skeletal Class I and Skeletal Class II in the MB group. However, differences in pharyngeal airway measurements were found between subjects with Skeletal Class I and those with Skeletal Class II in the NB group. This finding suggested that facial Skeletal anteroposterior classifications seem to have no association with the pharyngeal airway in subjects with MB mode. The difference in airway blockage levels between Class I and II patients in the MB group might be the cause of this outcome [30, 31]. According to Tourne, adenoid vegetation may reduce airway patency and induce postural adaptations at various levels of the pharyngeal airway. He asserted that the ultimate capacity of the pharynx is primarily determined by the growth and relative size of the adenotonsillar tissue than other factors [32].

Most of the previous studies that assessed the effect of anteroposterior relationship employed subjects with nasal breathing patterns [3, 7, 9]; for instance, Hakan et al. found that the airway volume was lower in participants with NB and Skeletal Class II than in those with NB and Skeletal Classes I and III [7]. Alves et al. [33] reported that the pharyngeal airway volume of individuals with NB and retruded mandibles was smaller than in those with a normal Skeletal pattern. Chan et al. [9] found a narrower nasopharyngeal airway in participants with Skeletal Class II than in those with other Skeletal patterns. According to a previous study by Nath et al., the pharyngeal airway volume was associated with the anteroposterior position of the mandible. Subjects with Skeletal Class II had a smaller airway volume than those with Skeletal Class I and III [34]. These findings are consistent with our finding that subjects with Skeletal Class II and NB had smaller airways than those with Skeletal Class I and NB. A variety of factors could be involved in this finding, such as the influence of retroposition of the mandible on the airway [35] and reduced maxillary transverse width [36]. Lee et al. [35] hypothesized that a reduction of the airway in patients with Skeletal Class II could result from mandibular and tongue posteroinferior movements. This possibility might explain the differences in pharyngeal airway between subjects with Skeletal Class II and those with Skeletal Class I in the NB group in the present study.

The current study showed that the pharyngeal airway volume and areas in subjects with NB were higher than those with MB in Skeletal Class I and II. These findings were consistent with the results of Alves et al. [23], who compared mouth and nasal breathing subjects. They discovered that the oropharyngeal airway volume and space were smaller in mouth breathing subjects than in nasal breathing subjects. Chung et al. [29] discovered that mouth breathing subjects had a smaller pharyngeal airway than nasal breathing. However, Chung et al. and Alves et al. did not classify subjects by anteroposterior relationship. As a result, their studies did not evaluate the sagittal pattern effect on the airway*.* A recent systematic review study by Zhao et al. showed that subjects with MB were associated with a reduction in the posterior airway spaces [2]. According to Tourné, adenoid hypertrophy and tongue mass might be essential factors in a reduction in the pharyngeal airway volume [32]. However, Kecik [37] thought that reduced pharyngeal airway in OSA and mouth breathing participants might be related to altered maxillary morphology in these subjects. This was also corroborated by Johal et al. They found that the difference in maxillary morphology between subjects with sleep disorders breathing and controls was the potential determinant that maxilla shape changes could be a potential etiology to the reduction of the pharyngeal airway in these subjects [38].

Besides the anteroposterior relationship, age has also been shown to affect airway size in some studies [8, 9]. The pattern of oropharyngeal soft tissue development was studied by Taylor et al. They noticed increased rapid growth at the posterior nasal spine to the pharyngeal wall and the posterior soft palate to the pharyngeal wall in two age groups (6–9 years and 12–15 years) as well as two stages of quiescence (9–12 years and 15–18 years) [39]. Another study on individuals aged 9–15 years showed consistent results [9]. These findings suggest that pharyngeal airway development in our patients aged 10–12 years might be stable, and aging might not have affected our results; therefore, our subjects were grouped in a single age group of 10–12 years.

Some dental characteristics of individuals with MB have been reported [15]. In the current study, MB subjects with Skeletal Classes I and Skeletal Classes II showed a reduced maxillary arch width. Aznar et al. [40] reported a reduction in the maxillary width at the canine level in individuals with MB. D’Ascanio et al. showed a reduction in the maxillary width at the first molar level. Harari et al. found constriction in the canine and first molar regions [15], which agreed with our study findings. Furthermore, in this study, the palatal area in individuals with MB was significantly smaller than in subjects with NB. In a previous study, authors reported that the MB habit caused Skeletal constriction of the maxilla, which altered the palatal surface and volume. The authors of this previous study also showed that the palatal surface area of participants with MB was reduced by 13.5% compared with that of participants with NB [17].

Similar to previous research, the current study showed that subjects with MB had a greater palatal height than those with NB [15]. Lione et al. showed that subjects with MB had a significantly greater palatal height in the level of first permanent molars and the second deciduous molars. They suggested that a deep palatal morphology was associated with a prolonged oral breathing habit [18].

In our research, Skeletal Class II subjects had significantly higher palatal height than Skeletal Class I individuals. According to Staley et al. [41], constriction of the maxillary arch is a compensatory mechanism of the maxilla for maintenance of occlusion when the mandible is retruded. Furthermore, subjects with Skeletal Class II had a smaller MWC than those with Skeletal Class I in this study. Previous studies had reported that the maxillary width was smaller in subjects with Skeletal Class II than in those with Skeletal Class I [21]. Nasal obstruction, a low tongue position, abnormal swallowing, and sucking habits contribute to a reduction in maxillary width in individuals with Skeletal Class II [41]. Nevertheless, a previous study showed no difference in the maxillary width between Skeletal Class I and II groups [19].This difference between studies might be due to differences in methodologies, measurement techniques, and race.

Even though cephalometric radiographs have a lower radiation dose than CBCT, CBCT is the preferred method for the convenience of 3D analysis. CBCT scans have been used to measure the pharyngeal airway and evaluate transverse changes following maxillary expansion in previous studies [42, 43]. Furthermore, CBCT has a lower radiation dosage than multislice computed tomography [20]. However, CBCT should be performed whenever required for only diagnostic reasons following as low as reasonably achievable principles [44]. Authors’ institution follows as low as reasonably achievable(ALARA) guidelines for all radiographic procedures. All retrospective CBCT scans used in this research were obtained for clinical reasons.

## Limitation

In our study, orthodontists and otolaryngologists worked together to diagnose mouth breathing. A thorough physical examination by an experienced otorhinolaryngologist included rhinomanometry, anterior rhinoscopy, flexible nasopharyngoscopy, or nasopharyngeal x-ray to evaluate nasal airflow and pressure while breathing, as well as pharyngeal airway obstruction. The otorhinolaryngologist and orthodontist categorized the participants as nasal or mouth breathers only after completing these examinations. However, we recommend a larger sample-sized study with a more objectively based method for classifying participants by breathing mode [45]. The second limitation was that the individuals with Skeletal Class III were not included in the current study because of the inadequate sample size. Future research should involve the relationship between the skeletal patterns (Classes I, II and III) with the breathing mode, pharyngeal airway, and maxillary arch. Furthermore, because of the smaller sample size, our sample size was not categorized by sex. Therefore we recommend future research with enough sample size to assess the influence of sex on the airway and maxillary arch.

The current study also has a study design limitation since it is a cross-sectional study in which the association does not suggest a causal relationship. As a result, future population-based longitudinal research is suggested.

## Conclusion

This study showed that children with MB might have smaller pharyngeal airway and maxillary arch dimensions but a greater palatal height than those with NB, regardless of the anteroposterior skeletal classification. Children with Skeletal Class I might have a larger maxillary width of the canines than those with Skeletal Class II in those with MB and NB. However, children with Skeletal Class II might have a greater palatal height than those with Skeletal Class I in those with MB and NB. The anteroposterior Skeletal classification appears to have no association with the pharyngeal airway size in children with MB. However, the anteroposterior skeletal classification might be associated with the pharyngeal airway in NB children; because NB subjects with Skeletal Class I showed larger pharyngeal airway measurements than those with Skeletal Class II.

## Data Availability

Data used and/or analyzed during the current study are available from the corresponding author upon request.

## Abbreviations

MB: Mouth breathing
NB: Nasal breathing
